# Collaborating with interventional pulmonology in managing a massive tracheoesophageal fistula that extends from cricoid to carina: a case report

**DOI:** 10.1186/s40981-017-0133-2

**Published:** 2017-12-04

**Authors:** Luis E. Tollinche, Mohit Chawla, Eunice W. Lee, A. Rolando Peralta

**Affiliations:** Memorial Sloan Kettering Cancer Center, 1275 York Avenue, C330F, New York, NY 10065 USA

**Keywords:** Tracheoesophageal fistula, Bronchoscopy, Tracheal stent, Esophageal stent, Jet ventilation

## Abstract

Tracheoesophageal fistulas (TEF) present a perioperative management challenge. A 62 year-old man with esophageal carcinoma presented with a large tracheoesophageal fistula extending most of the trachea. Previously, the patient had two overlapping esophageal and one tracheal stent placed, but he developed progressive tracheal disruption due to esophageal stent perforation near the level of the cricoid. This case describes the anesthetic management of tracheal stent placement for an expanding TEF. Management included a spontaneous breathing inhalation induction followed by ventilation through a supraglottic device—laryngeal mask airway (LMA). Finally, during rigid bronchoscopy, a combination of bag ventilation and jet ventilation was utilized.

## Background

The presence of a large tracheoesophageal fistula (TEF) poses an airway management challenge to the anesthesiologist. Despite an abundance of literature describing various techniques to mitigate the attendant risks during the perioperative period, there does not exist a consensus on the best approach for induction and maintenance of anesthesia [1, 2]. Myriad possibilities have been put forth and most providers have their preferred technique, but there is no evidence that one is superior to another [3]. It is known that positive pressure ventilation should be avoided whenever possible because air will pass through the point of least resistance—across the TEF and into the stomach [4]. The majority of literature dedicated to this topic centers on pediatric patients and management of congenital TEF. Less common are reports and studies citing management techniques for iatrogenic or acquired TEF in the adult population [5]. The use of laryngeal mask airway for insertion of a stent has been reported [6–8], and several techniques have been reported in the anesthesia literature including using a fogarty catheter to occlude the TEF during ventilation, intentional mainstem intubation to avoid ventilating across the TEF, and even using a guidewire to bypass a tracheal stricture and TEF [9–11]. The risks of anesthesia for adults and children with TEF overlap in many ways, but what is not known is the best technique for ventilating a patient with a large TEF [12].

## Case presentation

We present the case of a 62-year-old man former cigarette smoker with locally recurrent unresectable squamous cell carcinoma of the esophagus previously treated with concurrent chemotherapy and radiation. He presented with worsening cough, secretions, and halitosis due to malignant TEF. The TEF was managed by an outside institution with two sequential overlapping esophageal stents and subsequently one uncovered self-expanding metallic tracheal stent (18 mm) due to progressive extension of the fistula. Concurrently, he was receiving weekly carboplatin and paclitaxel for HPV-negative T3N2cM0 base of tongue squamous cell carcinoma.

**Fig. 1:**
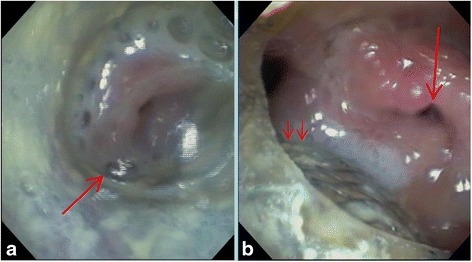
Supplemental Digital Content 1. **a**. Distal migration of previously placed metallic tracheal stent into right mainstem bronchus. **b** Consequent high-grade right mainstem bronchostenosis (single arrow); proximal revision of metallic tracheal stent reveals large tracheo-esophageal defect and esophageal stent

Upon presentation to our institution, bronchoscopy was performed by interventional pulmonology. This showed erosion of the esophageal stent into the trachea causing a large underlying 5-cm TEF extending from the proximal trachea to the main carina. The fistula was mostly covered by the noted esophageal stents; however, the tracheal stent had migrated into the right mainstem bronchus. We also noted a small mucosal ridge at the proximal edge of the fistula, approximately 4 cm below the cricoid. The migrated metallic tracheal stent was not amenable to removal as it would cause further airway injury and loss of seal of the majority of the fistula. Metallic stent was thus repositioned proximally to cover the proximal aspect of the fistula and the small mucosal ridge (see Fig. 1). To secure this stent in position and cover the remaining portions of the fistula, a telescoping Dumon Y-stent was placed via rigid bronchoscopy after balloon dilatation of the noted right mainstem bronchostenosis. This adequately covered the fistula.

**Fig. 2:**
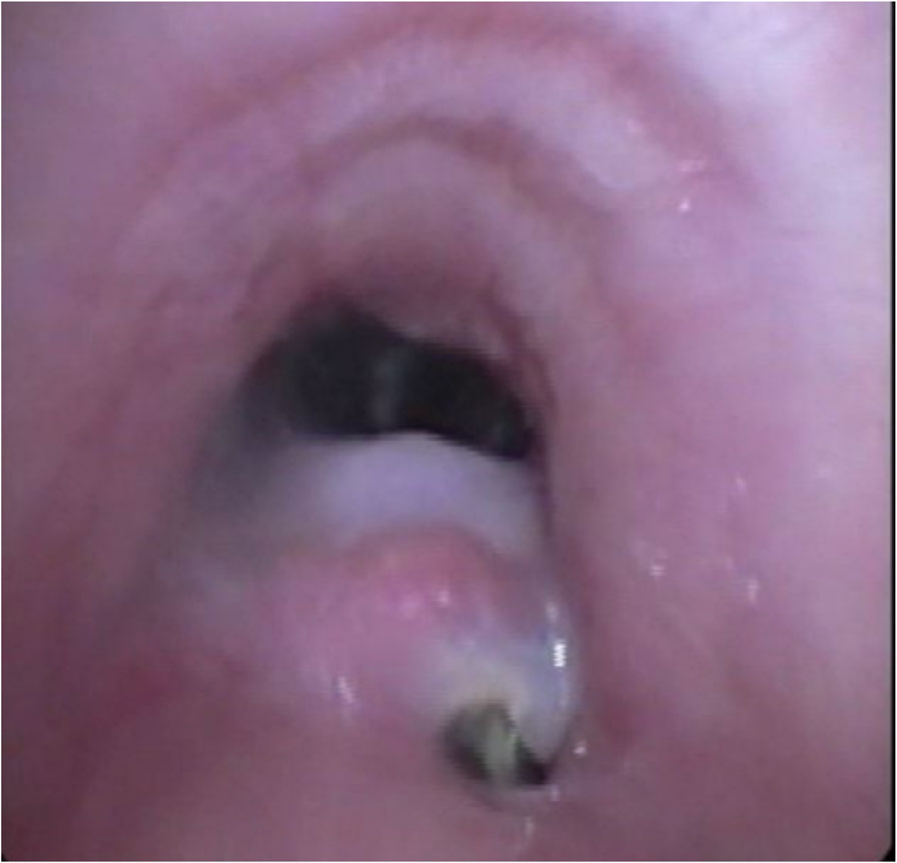
Supplemental Digital Content 2. Proximal partly obstructing granulation tissue, mucosal ridge, and new tracheoesophageal defect all due to adjacent esophageal stent

**Fig. 3:**
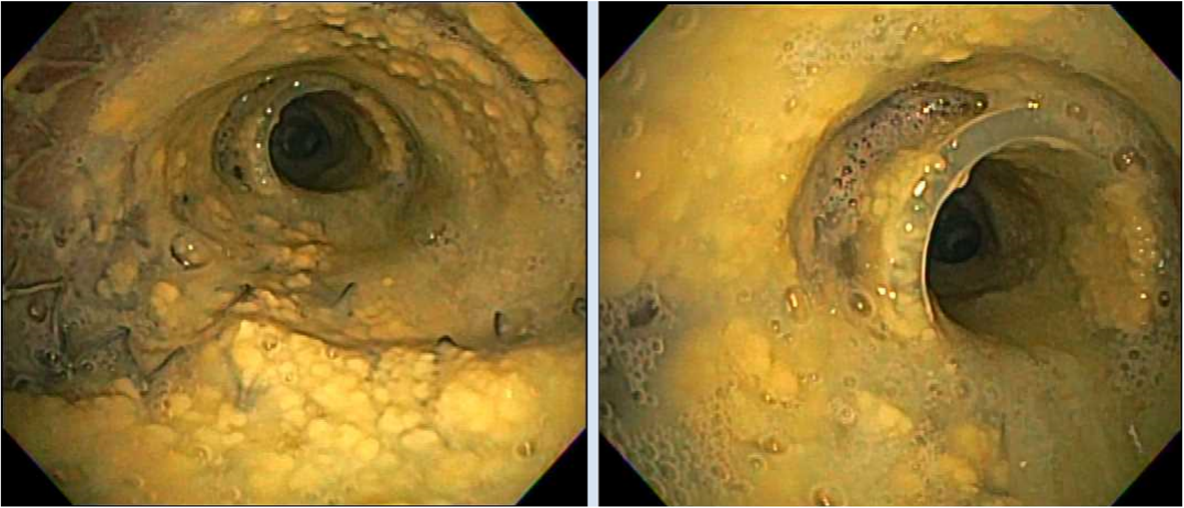
Supplemental Digital Content 3. In situ tracheal stents with infected inspissated secretions

The patient presented 1 month later for scheduled follow-up. He reported improvement in dyspnea and cough; however, worsening halitosis was noted. A flexible bronchoscopy was then performed for inspection and stent evaluation. Bronchoscopy revealed that both tracheal stents were in adequate position; however, we noted proximal granulation formation related to the metallic tracheal stent causing 50% obstruction of the airway and new extension of the TEF on the posterior tracheal wall due to erosion of the proximal esophageal stent (see Fig. 2). The lumen of this stent was easily visualized bronchoscopically. Both lesions were located just proximal to the tracheal metallic stent around 35 mm below the cricoid cartilage. We also noted copious mucopurulent secretions adherent to the silicone Dumon Y stent and in between the tracheal stents. These findings were consistent with stent infection and tracheobronchitis due to a gastrointestinal organism in the presence of a TEF (see Fig. 3).

After 2 weeks of antibiotic therapy (total planned course 4 weeks), we performed a rigid bronchoscopy to address the new TEF and proximal granulation. The patient was preoxygenated with the head of the bed elevated and the existing gastrostomy tube was placed to suction. After 5 min of preoxygenation, inhalation induction was initiated with 8% sevoflurane.

A propofol infusion was initiated concurrently and infused at rates 100–120 mcg/kg/min for the entire case—titrated initially to maintain spontaneous ventilation. The combination of sevoflurance and propofol was chosen because inhalational agent cannot be administered during rigid bronchoscopy—the patient would require total intravenous anesthesia during the rigid bronchoscopy. The patient breathed spontaneously throughout induction without positive-pressure assistance. A laryngeal mask airway (LMA size 4) was placed without difficulty, and flexible bronchoscopy was initiated. FiO2 100% at 3–10 L/min with sevoflurane 1% were maintained throughout the case, and oxygen saturations remained at 100%. An initial remifentanil bolus of 1 mcg/kg and infusion rate of 0.3 mcg/kg/min was administered.

After taking careful airway measurements, a Dumon tracheal stent was customized and loaded into the stent deployer. Upon completion of the flexible bronchoscopy, the remifentanil infusion was increased to 0.4 mcg/kg min and pt. became apneic. The LMA was removed and the rigid bronchoscope was inserted.

**Fig. 4:**
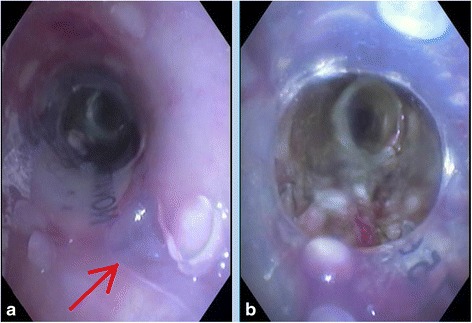
Supplemental Digital Content 4. Newly placed proximal silicon tracheal stent. **a**. Sealed proximal tracheoesophageal fistula. **b** Three telescoped tracheal stents

The interventional pulmonologist intubated the patient with a rigid bronchoscope (Bryan-Dumon series II 13.2 mm outer diameter tracheal length), and this was positioned within the proximal aspect of the metallic tracheal stent. Gentle bag ventilation over the side port of the bronchoscope was initiated; PIP averaged 11–12 cmH_2_O over the 17 min of rigid bronchoscopy. The chest was visually inspected for chest movement, and the PIP was titrated to the minimum PIP needed to ensure adequate chest excursion. Jet ventilation was started only after the TEF and granulation tissue were carefully bypassed. High-frequency jet ventilation, by increasing minute ventilation, has been shown to improve CO2 removal across a wide spectrum of frequencies and also to provide adequate oxygenation by increasing lung volume. Jet ventilation enhances technical access and improves ventilation during rigid bronchoscopic procedures [13]. Right before stent deployment, ventilation was momentarily held and the rigid bronchoscope was positioned in the desired location for stent. Via the rigid bronchoscope, a telescoping silicone Dumon tracheal stent (outer diameter 16 mm, customized length 45 mm) was placed overlapping the proximal aspect of the existing tracheal metallic stent by 15 mm and extending proximally up to 5 mm below the cricoid cartilage. This stent succeeded in both adequately covering the granulation and sealed the proximal TEF and successfully telescoped into the previously repositioned in situ metallic stent (see Fig. 4).

Direct visualization of the airway via flexible bronchoscopy did not reveal worsening tracheal disruption throughout the procedure. The LMA was replaced; propofol, remifentanil, and sevoflurane were discontinued; and the patient resumed spontaneous ventilations throughout emergence.

The patient tolerated the procedure well and no immediate complications were noted. Six weeks after the procedure, the patient was clinically stable.

## Conclusions

This case presented an airway and ventilation challenge as jet ventilation could worsen the tracheoesophageal fistula (TEF) and result in gastric overinflation. We were met with the usual challenges of TEF management but were further limited because of the location of our patient’s TEF. Given the location near the cricoid and the need to place a tracheal stent in the upper trachea, we exhausted the usual possibilities. The interventional pulmonologist required rigid bronchoscopy to correctly deploy the tracheal silicone stent, as additional self-expanding metallic stenting would have further extended the fistula. We were, therefore, unable to intubate with a conventional endotracheal tube or utilize a technique that had previously been successful for this patient—performing flexible bronchoscopy through a supraglottic airway. In order to decrease these risks, we connected the existing gastrostomy tube to wall suction and also minimized jet ventilation time by first using a supraglottic airway (i-gel, Intersurgical Inc., East Syracuse, NY) for inspection and bronchoscopic measurements. When we were ready for stent deployment, we utilized what many authors describe as second best option (after spontaneous ventilation) in ventilating a TEF. We performed “gentle bagging” by connecting our anesthesia circuit to the side port of the rigid scope. As soon as stent position was confirmed, we were able to jet ventilate more deliberately and complete the rigid bronchoscopy. Our goals in this case were consistent with the perioperative goals of all TEF cases—we wanted to provide general anesthesia and adequate ventilation to the lungs without ventilating the TEF. We were not able to utilize conventional techniques given the location and size of the TEF, so we implemented a stepwise sequence of maneuvers: (1) decompress stomach and apply continuous suction to the gastrostomy, (2) induce with spontaneous ventilation and perform flexible bronchoscopy via a supraglottic airway, (3) bag ventilate through side port of rigid bronchoscope until deployment of stent, and (4) judicious jet ventilation.

This case underscores the need for further investigation to define the best approach to managing TEF in adult patients [14]. Furthermore, most literature on this topic describes only TEF near or below the carina. To our knowledge, this is the first case report of successful rigid bronchoscopy for a TEF of this size and location.
